# Why restricting access to abortion damages women’s health

**DOI:** 10.1371/journal.pmed.1004075

**Published:** 2022-07-26

**Authors:** The PLOS Medicine Editors

**Affiliations:** Public Library of Science, San Francisco, California, United States of America

## Abstract

Dr. Caitlin Moyer discusses the implications, for women globally, of restricting access to abortion care.

In late June, the landmark *Roe v*. *Wade* ruling was overturned by the United States Supreme Court, a decision, decried by human rights experts at the United Nations [1], that leaves many women and girls without the right to obtain abortion care that was established nearly 50 years ago. The consequences of limited or nonextant access to safe abortion services in the US remain to be seen; however, information gleaned from abortion-related policies worldwide provides insight into the likely health effects of this abrupt reversal in abortion policy. The US Supreme Court’s decision should serve to amplify the global call for strategies to mitigate the inevitable repercussions for women’s health.

Upholding reproductive rights is crucial for the health of women and girls worldwide, and access to a safe abortion is central to this, yet policies in several countries either severely limit or actively prevent access to appropriate abortion care and services [2]. However, there is little to suggest that those countries and jurisdictions with abortion bans or heavily restrictive laws see fewer abortions performed. According to a modeling study of pregnancy intentions and abortion from the 1990s to 2019, rates of unintended pregnancies ending in abortion are broadly similar regardless of a country’s legal status of abortion, and unintended pregnancy rates are higher among countries with abortion restrictions [3]. Abortion is widely considered to be a low-risk procedure. Abortion-related deaths most likely occur in the context of unsafe abortion practices and are reported to account for 8% (95% UI 4.7–13.2%) of maternal deaths [4], making them a top direct contributor to maternal deaths globally, alongside hemorrhage, hypertension, and sepsis. Restrictive abortion policies may not lower the overall rates of abortion, but they can drive increasing rates of unsafe abortions, as women resort to seeking abortions covertly. Such abortions are often performed by untrained practitioners or involve harmful methods. Perhaps unsurprisingly, most abortions that take place in countries with restrictive abortion access policies are not considered safe [5], potentially contributing to maternal morbidity and mortality. A study of 162 countries found that maternal mortality rates are lower in countries with more flexible abortion access laws [6], suggesting that changes in abortion policies could have grievous implications for maternal deaths.

It is not yet known if the reneging of federal protection of abortion rights will impact maternal deaths in the US; however, in the years following the 1973 *Roe v*. *Wade* decision, numbers of reported deaths associated with illegal abortions, defined as those performed by an unlicensed practitioner, declined, hovering between zero and 2 deaths from the 1980s to 2018, down from 35 in 1972 [7] and 19 reported in 1973 [8]. It is possible that limits on access to timely and safe abortion care could drive this number back up and add to the already unacceptably high maternal mortality rate in the US, potentially exacerbating the persistent disparities in maternal mortality based on socioeconomic deprivation, race and ethnicity, and other factors [9].

Legal and social barriers that impede access to safe abortions are detrimental to the health and survival of women and girls; thus, constructing policies ensuring access to safe abortion services should be an urgent priority. Placing undue hurdles between women and access to abortion care is associated with undesirable health outcomes. For example, a 2011 change to medication abortion laws in one US state that involved increased medication costs and restricted the timing and location where abortion services could be provided was associated with an increase in rates of women requiring additional medical interventions [10]. Lending international weight to this argument, dissolution of barriers to safe abortion access was emphasized in the March 2022 update of WHO guidance on abortion care [11], echoing a 2018 comment on the International Covenant on Civil and Political Rights released by the United Nations Human Rights Committee [12] that called on member states to remove existing barriers and not enact new restrictions on provision of safe abortion services so that pregnant women and girls do not need to turn to unsafe abortions.

In jurisdictions where prohibitive policies exist, more could be done to counter the impacts of new barriers by changing how abortion care is delivered and increasing accessibility. Protocols for the safe self-management of abortion can be implemented alongside provision of information and provider support. WHO guidance [11] suggests expanding the breadth of practitioners authorized to prescribe medical abortions to include nurses, midwives, and other cadres of healthcare workers. The guidelines also mention telemedicine as an approach to circumvent obstacles to seeking safe abortion services [11]. For those with access to the necessary technology, telemedicine services together with self-management of medication abortion can overcome travel-related barriers and ensure the privacy of those seeking treatment. Demands for telehealth services increased during the COVID-19 pandemic, and, according to one study, remote provision of abortion services in the US may be a promising option to counteract barriers and facilitate access [13].

In 2022, restrictive policies or outright bans on abortion services are discriminatory against women, obstructing their right to maintain autonomy over their own sexual and reproductive health. A post-*Roe* legal landscape that renders abortion more difficult or impossible to obtain safely will exacerbate an increasingly bleak picture of maternal health in the US; however, the US is just one example where increased effort is needed to overcome barriers to improving women’s healthcare. The reality is that such barriers continue to represent a threat to the health of women worldwide. Evidence-based changes to policy and practice that break down barriers and build new roads are required to enable women to access the healthcare they need.
